# Behavioral counseling to prevent childhood obesity – study protocol of a pragmatic trial in maternity and child health care

**DOI:** 10.1186/1471-2431-12-93

**Published:** 2012-07-03

**Authors:** Taina Mustila, Päivi Keskinen, Riitta Luoto

**Affiliations:** 1 Seinäjoki Central Hospital, Hanneksenrinne 7, 60220 Seinäjoki, Finland; 2 Vaasa Central Hospital, Hietalahdenkatu 2 – 4, 65130 Vaasa, Finland; 3 Pediatric Research Centre, 33014 University of Tampere, Tampere, Finland; 4 Tampere University Hospital, 33521, Tampere, Finland; 5 UKK Institute for Health Promotion, 33501, Tampere, Finland; 6 National Institute for Health and Welfare, 00271, Helsinki, Finland

**Keywords:** Childhood obesity, Intervention, Lifestyle, Pragmatic, Prevention

## Abstract

**Background:**

Prevention is considered effective in combating the obesity epidemic. Prenatal environment may increase offspring's risk for obesity. A child starts to adopt food preferences and other behavioral habits affecting weight gain during preschool years. We report the study protocol of a pragmatic lifestyle intervention aiming at primary prevention of childhood obesity.

**Methods/Design:**

A non-randomized controlled pragmatic trial in maternity and child health care clinics. The control group was recruited among families who visited the same clinics one year earlier. Eligibility criteria was mother at risk for gestational diabetes: body mass index ≥ 25 kg/m^2^, macrosomic newborn in any previous pregnancy, immediate family history of diabetes and/or age ≥ 40 years. All maternity clinics in town involved in recruitment. The gestational intervention consisted of individual counseling on diet and physical activity by a public health nurse, and of two group counseling sessions. Intervention continues until offspring’s age of five years. An option to participate a group counseling at child’s age 1 to 2 years was offered. The intervention includes advice on healthy diet, physical activity, sedentary behavior and sleeping pattern. The main outcome measure is offspring BMI z-score and its changes by the age of six years.

**Discussion:**

Early childhood is a critical time period for prevention of obesity. Pragmatic trials targeting this period are necessary in order to find effective obesity prevention programs feasible in normal health care practice.

**Trial registration:**

Clinical Trials gov NCT00970710

## Background

Obesity remains a worldwide costly health concern although the increase in prevalence of childhood obesity seems to be abating at least in some western countries [1,2]. Childhood obesity has several adverse outcomes during childhood, and since it often continues to adulthood, it increases cardiovascular morbidity among other health concerns in adult life [3,4]. Genetic susceptibility is a strong determinant for risk of being an obese child, but environment already starts to play a role during fetal life, infancy and preschool years [5-8] Once a child is obese it is difficult to reverse this adverse metabolic state with interventions [9]. This supports the rationale to find early preventive interventions. Since childhood obesity is a common health problem and concerns the whole community, the preventive methods should be applicable to normal health care practice and be cost-effective enough. Yet there are only few reported early interventions to prevent childhood obesity, and these are mostly not implemented in normal primary care practice. Many of these interventions especially those targeted at multiple factors affecting energy expenditure have resulted in some positive effects in weight development, but follow-up times have been short [10,11].

There is evidence that offspring of mothers gaining excessive weight and having impaired glucose tolerance during pregnancy are at higher risk for obesity [12-15]. Rapid weight gain in infancy and during preschool years is also a known risk factor for later obesity [8,16]. Breastfeeding may protect against obesity especially if the mother is overweight [17]. Parents have critical role in introducing healthy dietary habits to their offspring and food preferences often develop during preschool years [18]. Even preschool aged children spend a significant time in sedentary activities. Less physically active children are prone to obesity [8,19]. Reducing sedentary time has potential to mitigate childhood obesity risk [20]. Short sleep duration has been associated with greater risk for childhood obesity [21]. To reverse the obesity epidemic early intervention programs are thought to be an opportunity. An appropriate setting for these interventions would be municipal maternity and child health clinics, making it possible to reach the target population at moderate cost. Targeting preventive means at a risk group the cost-effectiveness of the intervention is presumed to be higher. The aim of this pragmatic multifaceted intervention programme is to prevent obesity in preschool children. In this paper we describe the protocol of the study.

## Methods

### Study objectives

The primary aim is to evaluate whether a multifaceted structured lifestyle intervention in primary care setting has potential to prevent overweight among preschool children belonging to a risk group. Additional aims include assessing changes in metabolic profile, waist circumference, blood pressure, dietary habits and time spent physically active, sleep and screen time of the offspring.

### Hypothesis

Participant intervention group mothers at risk of gestational diabetes gain less excess weight and have better glucose tolerance during pregnancy via intensified counseling on healthier diet and physical activity. These goals are expected to favour normal offspring weight development. Continued counseling in child health care clinics concerning age-appropriate diet, physical activity, sedentary behaviour and sleep pattern is expected to further diminish offspring’s risk for obesity.

### Study design and setting

The study (VACOPP Study = VAasa Childhood Obesity Primary Prevention Study) is non-randomized controlled clinical pragmatic trial. The setting is eight municipal maternity and 14 child health care clinics in city of Vaasa in western Finland. All maternity and child health care clinics in the city participated in recruiting and intervention. The cohort of mothers who gave birth year 2008 and had risk factors for developing GDM and their offspring were the targets for recruiting the control group. Mothers pregnant during 2009–2010 with the same risk factors were targetted for the intervention group. The control group mothers were recruited retrospective, before their children had reached one year of age. Ethical approval for the study was granted by the Ethics Committee of Vaasa Hospital District.

### Participants and recruitment

Informed written consent was provided by all participant mothers prior baseline assessments. The participants were mothers living in the city of Vaasa and belonged to a risk group for developing GDM. Criteria for belonging to this risk group were body mass index (BMI) ≥ 25 kg/m^2^, macrosomic newborn (weight ≥ 4500 g) in any previous pregnancy, immediate family history of diabetes and/or age ≥ 40 years. This group of mothers are offered oral glucose tolerance test (OGTT) during pregnancy by the municipal maternity care. The offspring of these mothers are the target children. The exclusion criteria for the mothers were: multiple pregnancies, inability to speak Finnish, substance abuse or psychiatric illness fundamentally affecting ability to function. Recruitment of the control group was performed via telephone calls by a research nurse asking permission to send written research information and consent forms to subjects’ homes. The intervention group recruitment took place in maternity clinics by public health nurses (PHN) at first contact. PHN gave the written research information and consent form. All mothers recruited were offered an opportunity to ask questions concerning the trial by telephone or e-mail of either the research nurse or the researchers. Since the study was a pragmatic trial, power calculations were not given priority. Due to the small number of expected participating mothers and retrospective control group the study would not reach statistical significance in a rigorous sense. The mean BMI z-score in the control group would be only a rough estimate thus making power calculations inaccurate [22].

### Intervention in maternity health care clinics

The intervention started in the maternity clinics and continues in child health care clinics until the child is five years old. The intervention is intensified multifaceted lifestyle counselling. The intervention group mothers were offered two group counseling sessions: one during both first and second trimester of pregnancy. 1.5 hour sessions were given by a trained physiotherapist and dietician employed in public health centre. Information on diet was according to the nutrition recommendations of Ministry of Social Affairs and Health during pregnancy especially concerning appropriate energy content, fibre, quality of carbohydrates and fat [23]. The physiotherapist gave information about suitable and sufficient amount of exercise during pregnancy [24], and the mothers participated in brief session of gymnastics suitable to do at home. The mothers also received information on the effect of healthy diet, exercise and appropriate weight gain during pregnancy on risk of having GDM, offspring’s perinatal problems and obesity risk. Written educational material on healthy diet and physical activity during pregnancy was also given. This counseling was repeated more briefly by the PHNs during 13 routine visits to maternity clinics. After delivery the mothers were given a written information leaflet by a PHN reminding them that breastfeeding up to 6 months of age is also recommended for appropriate weight gain of the infant.

### Intervention in child health care clinics

The families are offered to participate a 1.5 hour structured group counseling session when the child is 1 to 2 years old. It is given by physiotherapist and dietician employed in health care center and includes healthy diet recommendations to children under school age [23], as well as advice on suitable physical exercise at that age. The physical activity recommendations were according to those of Ministry of Social Affairs and Health [25]. Intervention group children are given a 30 to 60 minutes longer appointment with a PHN in child health care clinic at routine yearly control visits at 1, 2, 3, 4 and 5 years of age [Table 1. During these visits counseling on healthy diet and amount of physical exercise/day is given as well as advice about age-appropriate sleep and screen time. These issues are addressed with help of the Finnish Heart Association’s “The Smart Family- exercise and nutrition guidance method” [Additional file 1. This method includes an interview card, a folder with e.g. informative images to be studied together with the family, and a separate information folder for PHNs. With the help of the card the family estimates its own habits regarding lifestyle and it functions as a tool for motivational interview. The folder contains motivational pictures and written information about healthy diet and physical activity. The PHNs in both maternity and child health care were trained in three separate group sessions for the intervention group intensified lifestyle counseling, and thereafter reminded about the intervention protocol at least once yearly by e-mail. The training of PHNs to use The Smart Family- exercise and nutrition guidance method was performed by Finnish Heart Association’s educator in a whole day training session. The control group is getting the usual counseling used in Finnish maternity and child health care centers. 

**Table 1:** Timing of the intensified counseling in maternity and child health care clinics

***Intervention during pregnancy***						
**Gestational weeks 10-17**	**Gestational weeks 18-28**		**Gestational weeks 20-32**		***Gestational weeks 28-40***	
Counseling session given by a trained physiotherapist and dietician 1,5 hours and 2 routine visits to PHN with intensified counseling concerning diet and physical activity	3 routine visits to PHN with intensified counseling concerning diet and physical activity		Counseling session given by a trained physiotherapist and dietician 1,5 hours		8 routine visits to PHN with intensified counseling concerning diet and physical activity	
***Intervention in child health clinics***						
**0-6 months**	**1 year**	**1-2 years**	**2 years**	**3 years**	**4 years**	***5 years***
Information leaflet on breastfeeding	*Intensified counseling by PHN at child health care centre with “The Smart Family-exercise and nutrition guidance method”*	*Intensified counseling by PHN at child health care centre with “The Smart Family-exercise and nutrition guidance method”*	*Intensified counseling by PHN at child health care centre with “The Smart Family-exercise and nutrition guidance method”*	*Intensified counseling by PHN at child health care centre with “The Smart Family-exercise and nutrition guidance method”*	*Intensified counseling by PHN at child health care centre with “The Smart Family-exercise and nutrition guidance method”*	*Intensified counseling by PHN at child health care centre with “The Smart Family-exercise and nutrition guidance method”*

### Measures

The primary outcomes of the intervention are difference and changes in BMI z-scores among the study group offspring, and the proportion of children being overweight according to the new Finnish cut-off-values for (percentile curve passing through BMI 25 kg/m^2^) or obese (percentile curve passing through the BMI 30 kg/m^2^) [26]. PHNs measure children’s weight and length/height at 4 and 6 months of age and thereafter at 1, 2, 3, 4, 5 and 6 years of age.The final primary outcome measure time point will be at six years of age.

The secondary outcomes are duration of breastfeeding, frequency of offspring dietary intake of sugary beverages, pastry, sweets, fruit, berries, vegetables, type butter or margarine used and frequency of eating takeaway food as well as eating pattern are recorded yearly. Daily average screen and sleep time are recorded. Waist circumference (WC) and blood pressure (BP) are measured at 2, 3, 4, 5 and 6 years of age. Metabolic markers (fasting triglycerides, HDL-cholesterol, glucose, insulin and alaninaminotransferase) are measured at 2, 4 and 6 years of age.

### Data collection

Mothers weight gain, BP, sleep duration, mother’s own estimate about weekly physical exercise during pregnancy, results of 2-hour OGTT at 27–28 weeks of pregnancy were recorded in questionnaires filled in partly by the PHN and partly by the mothers once during the first, second and third trimester of pregnancy. The PHN measured and wrote down the physical measures in the questionnaires except for the control group’s measures during pregnancy, which were filled in by the mothers themselves. The mothers transferred this data to study questionnaire from their maternity card, which was filled in by a PHN during pregnancy. The offspring data is recorded in questionnaires, which are filled in at the yearly appointment with the PHN. Long term illnesses affecting growth are recorded. The PHNs measure the length/height, weight, BP and WC of the child and add them to the questionnaire. The children’s weight is measured to the nearest 0.01 kg without clothes until one year of age, and thereafter to the nearest 0.1 kg on a standard electronic scale with light clothing. Children under 2 years are measured in recumbent position and thereafter in standing position to the nearest millimetre with a standard stadiometer. WC is instructed to be measured on the midpoint between the lower costal border and the iliac crest. BP is measured by PHN using an automated BP monitor (Omron M6) under standard conditions with two repeated measurements. The children’s dietary intakes are recorded yearly by questionnaires filled by mother or father of the child, and are measured as average consumption/day or week. The children’s eating patterns are also recorded. The time children spend physically active is measured as parents estimate of time spent outdoors, moderate intensity or structured physical activity time as hours/day or week. Daily average screen time and sleep time are measured as parents’ estimate yearly. Fasting blood samples are collected in the local medical laboratory used by health centre in Vaasa (Vaasa Central Hospital) and analysed using standard automated techniques. Laboratory results are recorded by the researcher directly from the laboratory score sheet. The timing of questionnaires, physical measurements and laboratory tests are listed in Table 2.

**Table 2:** Timing of questionnaires, physical measurements and laboratory tests

**Maternity care**	**Weeks' gestation**			
	**8-12**	**26-28**	**37**	**After childbirth**
	Mother's age	Mother´s average	Mother´s average sleep h/d	Gestation week of childbirth
	Mother's education	sleep h/d	Mother's average physical activity	Birthweight
	Father’s	Mother's average	h/d	Birth length
	education	physical activity h/d	Blood pressure	Birth head circumference
	Mother's weight and height before pregnancy	Blood pressure	Mother's weight	
	Mothers chronic illness	Oral glucose		
	Fathers weight and height	tolerance test		
	History of GDM			
	History of newborn > 4500 g			
	Smoking during pregnancy			
	Previous deliveries			
	Immediate family history of DM 2, CAD, hypertension, obesity and hypercholesterolemia			
	Mother´s average sleep h/d			
	Mother's average physical activity h/d			
	Blood pressure			
**Child health care**	**1 year of age**	2, 4 and 6 years of age *	**3 and 5 years of age**	
	Chronic illness	Chronic illness	Chronic illness	
	4 and 6 months weight and length	Consumption of	Consumption of beverage, fruits, vegetables, berries, sweets,	
	Weight	beverage, fruits,	pastry, bread, yoghurt, eating at takeaway restaurant	
	Length	vegetables, berries,	Regularity of meals	
		sweets, pastry, bread,	Sleep time h/d	
		yoghurt, eating at	Daily physical activity/outdoor activities	
		takeaway restaurant	Daily screentime	
		Regularity of meals	Weight	
		Sleep time h/d	Height	
		Daily physical	Waist circumference	
		activity/outdoor	Blood pressure	
		activities		
		Daily screentime		
		Weight		
		Height		
		Waist circumference		
		Blood pressure		
		fP-glucose		
		fP-insulin		
		fP-cholesterol		
		fP-HDL-cholesterol		
		fP-triglyserides		
		P-ALAT		

* At these timepoints the parents are offered to have their weight, waist circumference, blood pressure, fP-glucose, fP-cholesterol, fP-HDL-cholesterol and fP-triglycerides measured by health care centre; fP = fasting plasma; GDM = gestational diabetes mellitus; DM = diabetes mellitus; CAD = coronary artery disease; P-ALAT = plasma alaninaminotransferase.

### Statistical analysis

Characteristics of the study participants will be described using means and standard deviations or frequencies and proportions. The child’s size during follow-up will be analysed using weight and length/height converted to BMI (weight (kg)/height (m^2^))-for-age and weight-for-length/height and their SDSs (z-scores) according to the recently updated Finnish growth reference (26). Exact age of the child will be used in all growth analyses. Mixed-effects linear regression models will be used to analyse the association of weight-for-length/height z-score and BMI z-score over time by group (intervention/control). These models allow for a difference between groups at baseline, linear changes of z-score over time and the difference of improvement between groups, which can be viewed as the intervention effect (i.e. interaction term). The goodness-of-fit of the models will be evaluated visually by normal probability and residual plots and also tested by the normality of the residuals (Kolmogorov-Smirnov test). All analyses will be performed using STATA software (version 12.0 for Windows), StataCorp LP, Texas, USA.

## Discussion

This pragmatic lifestyle intervention study aims to reduce the risk of obesity among a selected risk group of preschool children. It is based on following components: intensified diet and physical activity counseling during pregnancy; advicing mothers to favour breastfeeding and mother and father to help their child to adopt healthy food preferences; advicing parents to encourage their children to be physically active, minimizing sedentary activity time, and also reminding parents about the role of an appropriate amount of sleep in weight development. The intervention is still ongoing and the final feasibility and effectiveness of this trial will be assessed at offspring age of six years, but we will report also earlier preliminary results.

Pragmatic trials are considered to have greater external validity than do explanatory studies, but instead the internal validity is considered to be lower [27]. Our study was designed to be integrated in routine health care practice and to maximize the applicability of results to usual care setting. Practitioners who perform the intervention may vary in the way they deliver the planned counseling and the compliance of both practitioners and families may be variable. This results in weaker internal validity. For the internal validity randomization is considered the best way to select the trial participants also in pragmatic trials [27]. However, conducting a randomized controlled trial is not always feasible, and the randomization process may reduce willingness to participate in the trial especially in lifestyle interventions, where problems in recruiting enough participants are usual. Case control study design is considered the second best design in intervention studies when randomization is not feasible, especially when the study groups are matched for the characteristics that may affect the result [28]. Raaijmakers M et al. assessed randomized vs. non-randomized study with group matching design in their intervention and found that non-randomized appropriate matching resulted in 34% of their simulated trials in a more equally balanced distribution of their key characteristics compared with randomization, thus suggesting acceptability of non-randomized trials when the key characteristics are equally balanced in the groups [29]. It has also been stated that even retrospective recruitment could be used in primary care settings when non-acute conditions are studied [30]. In retrospective recruitment randomization is not possible and may make participant flow irregular, as it does in our study, too. The advantages of retrospective recruitment are that it shortens the recruitment time and reduces practical staff workload. Disadvantages of retrospective recruitment include weaker control of bias or other unknown factors influencing the effectiveness of the trial.

There are several methodological limitations in our study. We did not use randomizing and recruited the control group retrospectively in order to get a larger sample size for our trial planned to be performed in only one city and one risk group. The rationale behind this group selection was that it made the recruitment period shorter, the health centre staff’s workload lower in recruiting, and we were able to recruit larger study groups in this small city willing to participate this long-term intervention study. Randomization is not possible when the control group is retrospectively recruited, but because motivation to participate trials is a problem, especially when lifestyle intervention is involved, the randomization could have further reduced families’ willingness to participate in the study. However, we estimated that the groups would be comparable being families living in the same city and recruited from the same population. We also assumed that the one-year retrospective control group would not induce bias in the results since there were no major changes in municipal health care practices during that period. The retrospective control group could however cause bias, because the same PHNs who perform the intervention take care of the usual counselling practice of the control group in child health care clinics, but not in maternity clinics.

The strength of this study is that is designed to be a pragmatic trial integrated in health care practice thus having good prospect to be a sustainable part of municipal health care. The intervention costs are low since the existing clinical staff are the intervention practitioner. The follow-up time will be up to six years of offspring’s age thus providing fairly long-term follow-up. Our intervention targeted several lifestyle factors that are known to affect the child’s weight gain. Multifaceted intervention programmes are thought to be suitable for pragmatic trials and most effective in preventing overweight, since obesity is a result of many lifestyle factors in addition to genetic susceptibility. Moreover our intervention already starts during pregnancy and is planned to continue until the offspring is five years of age. This longer duration gives the intervention better chances of resulting in healthy weight gain. We are targeting mothers known to be at risk of having overweight or obese offspring. The positive intervention effect is more probable than in a trial targeting a population without specifically sought risk characteristics. We also offered the parents a chance to monitor their own weight, BP, WC and metabolic markers, which we thought would motivate the parents to continue in the study, and also to function as a public health promotive act by helping to find parents at risk of cardiovascular diseases.

## Conclusion

Obesity originates prenatally and in early childhood. If overweight is already gained during the preschool years, the risk of becoming an obese adult is high. Thus preventive means should start early. The importance of pragmatic trials is that they help to define the best use of limited resources as well as policymakers and practitioners to make choices between customary care and the new counseling practice. Attempts to achieve methodological purity in explanatory trials can produce results that are not applicable in real life, but attempts to achieve full generalizability may also yield unreliable results. Pragmatic trials are not planned to study the contributors of its different components to the results and they should have long-term follow-up to ascertain whether the possible benefits are sustainable (27). Our intervention has potential to be integrated in routine municipal maternity and child health care practice with moderate costs to society.

## Abbreviations

BMI, Body mass index; GDM, Gestational diabetes mellitus; OGTT, Oral glucose tolerance test; PHN, Public health nurse; WC, Waist circumference; BP, Blood pressure.

## Competing interests

The authors declare that they have no competing interests.

## Authors’ contributions

TM and PK contributed to conception and design of the study. TM, RL and PK participated in drafting and revising the manuscript. All authors read and approved the final version of the manuscript.

## Pre-publication history

The pre-publication history for this paper can be accessed here:

http://www.biomedcentral.com/1471-2431/12/93/prepub

## Supplementary Material

Additional file 1 Information on “the Smart Family”-exercise and nutrition guidance method. Click here for file

## References

[B1] De Onis M Blössner M Borghi E Global prevalence and trends of overweight and obesity among preschool children Am J Clin Nutr 2010 92 1257 1264 10.3945/ajcn.2010.29786 20861173

[B2] Olds T Maher C Zumin S Péneau S Lioret S Castetbon K Bellisle De-Wilde J Hohepa M Maddison R Lissner L Sjöberg A Zimmermann M Aeberli I Ogden C Flegal K Summerbell C Evidence that the prevalence of childhood overweight is plateauing: data from nine countries Int J Pediatr Obes 2011 6 342 360 10.3109/17477166.2011.605895 21838570

[B3] Han JC Lawlor DA Kimm SY Childhood obesity Lancet 2010 375 1737 1748 10.1016/S0140-6736(10)60171-7 20451244 PMC3073855

[B4] Juonala M Magnussen CG Berenson GS Venn A Burns TL Sabin MA Srinivasan SR Daniels SR Davis PH Chen W Sun C Cheung M Viikari JS Dwyer T Raitakari OT Childhood adiposity, adult adiposity, and cardiovascular risk factors N Engl J Med 2011 365 1876 1885 10.1056/NEJMoa1010112 22087679

[B5] Dabalea D Crume T Maternal environment and the transgenerational cycle of obesity and diabetes Diabetes 2011 60 1849 1855 10.2337/db11-0400 21709280 PMC3121421

[B6] Lawlor DA Lichtenstein P Långström N Association of maternal diabetes mellitus in pregnancy with offspring adiposity into early adulthood. Sibling study in a prospective cohort of 280 866 men from 248 293 families Circulation 2011 123 258 265 10.1161/CIRCULATIONAHA.110.980169 21220735 PMC4440894

[B7] Ong KK Loos RJ Rapid infancy weight gain and subsequent obesity:systematic reviews and hopeful suggestions Acta Paediatr 2006 95 904 908 10.1080/08035250600719754 16882560

[B8] Reilly JJ Armstrong J Dorosty AR Emmett PM Ness A Rogers I Steer C Sherriff A Early life risk factors for obesity in childhood: cohort study BMJ 2005 330 1357 10.1136/bmj.38470.670903.E0 15908441 PMC558282

[B9] Oude Luttikhuis H Baur L Jansen H Shrewsbury VA O'Malley C Stolk RP Summerbell CD Interventions for treating obesity in children Cochrane Database Syst Rev 2009 21 CD001872 19160202 10.1002/14651858.CD001872.pub2

[B10] Monasta L Batty GD Macaluso A Ronfani L Lutje V Bavcar A Interventions for the prevention of overweight and obesity in preschool children: a systematic review of randomized controlled trials Obes Rev 2011 12 e107 118 10.1111/j.1467-789X.2010.00774.x 20576004

[B11] Waters E de Silva-Sanigorski A Hall BJ Brown T Campbell KJ Gao Y Armstrong R Prosser L Summerbell CD Interventions for preventing obesity in children Cochrane Database Syst Rev 2011 7 CD001871 22161367 10.1002/14651858.CD001871.pub3

[B12] Dubois L Girard M Early determinants of overweight at 4.5 years in a population-based longitudinal study Int J Obes (Lond) 2006 3 610 617 16570091 10.1038/sj.ijo.0803141

[B13] Metzger BE Lowe LP Dyer AR Trimble ER Chaovarindr U Coustan DR Hadden DR McCance DR Hod M McIntyre HD Oats JJ Persson B Rogers MS Sacks DA The HAPO Study Cooperative Research Group Hyperglycemias and adverse pregnancy outcomes N Engl J Med 2008 358 1991 2002 18463375 10.1056/NEJMoa0707943

[B14] Fraser A Tilling K Macdonald-Wallis C Sattar N Brion MJ Benfield L Ness A Deanfield J Hingorani A Nelson SM Smith GD Lawlor DA Association of maternal weight gain in pregnancy with offspring obesity and metabolic and vascular traits in childhood Circulation 2010 121 2557 2564 10.1161/CIRCULATIONAHA.109.906081 20516377 PMC3505019

[B15] Wrotniak BH Shults J Butts S Stettler N Gestational weight gain and risk of overweight in the offspring at age 7 y in a multicenter multiethnic cohort study Am J Clin Nutr 2008 21 521 526 10.1093/ajcn/87.6.1818 18541573

[B16] Lagström H Hakanen M Niinikoski H Viikari J Rönnemaa T Saarinen M Pahkala K Simell O Growth patterns and obesity development in overweight or normal-weight 13-year-old adolescents: The STRIP Study Pediatrics 2008 122 e876 883 10.1542/peds.2007-2354 18829786

[B17] Buyken AE Karaolis-Danckert N Remer T Bolzenius K Landsberg B Kroke A Effects of breastfeeding on trajectories of body fat and BMI throughout childhood Obesity (Silver Spring) 2008 16 389 395 10.1038/oby.2007.57 18239649

[B18] Jones LR Steer CD Rogers IS Emmett PM Influences on child fruit and vegetable intake: sociodemographic, parental and child factors in a longitudinal cohort study Public Health Nutr 2010 13 1122 1130 10.1017/S1368980010000133 20196909

[B19] Jiménez-Pavón D Kelly J Reilly JJ Associations between objectively measured habitual physical activity and adiposity in children and adolescents: Systematic review Int J Pediatr Obes 2010 5 3 18 10.3109/17477160903067601 19562608

[B20] te Velde SJ van Nassau F Uijtdewilligen L van Stralen MM Cardon G De Craemer M Manios Y Brug J Chinapaw MJ Energy balance-related behaviours associated with overweight and obesity in preschool children: a systematic review of prospective studies Obes Rev 2012 13 Suppl 1 56 74 22309065 10.1111/j.1467-789X.2011.00960.x

[B21] Landhuis CE Poulton R Welch D Hancox RJ Childhood sleep time and long-term risk for obesity: a 32-year prospective birth cohort study Pediatrics 2008 122 955 960 10.1542/peds.2007-3521 18977973

[B22] Schulz KF Grimes DA Sample size calculations in randomized trials: mandatory and mystical Lancet 2005 365 1348 1353 10.1016/S0140-6736(05)61034-3 15823387

[B23] Hasunen K Kalavainen M Keinonen H Lagström H Lyytikäinen A Nurttila A Peltola T Talvia S The Child, Family and Food. Nutrition recommendations for infants and young children as well as pregnant and breastfeeding mothers 2004 Publications of the Ministry of Social Affairs and Health, Helsinki

[B24] Aittasalo M Pasanen M Fogelholm M Kinnunen TI Ojala K Luoto R Physical activity counselling in maternity and child health care – a controlled trial BMC Womens health 2008 8 14 10.1186/1472-6874-8-14 18702803 PMC2527301

[B25] Recommendations for physical activity in early childhood education Handbooks of the Ministry of Social Affairs and Health 2005, Helsinki

[B26] Saari A Sankilampi U Hannila ML Kiviniemi V Kesseli K Dunkel L Finnish growth references for children and adolescents aged 0 to 20 years: Length/height-for-age, weight-for-length/height, and body mass index-for-age Ann Med 2011 43 235 248 10.3109/07853890.2010.515603 20854213

[B27] Godwin M Ruhland L Casson I MacDonald S Delva D Birtwhistle R Lam M Seguin R Pragmatic controlled clinical trials in primary care: the struggle between external and internal validity BMC Med Res Methodol 2003 3 28 10.1186/1471-2288-3-28 14690550 PMC317298

[B28] Society for Prevention Research Standards of evidence: criteria for efficacy, effectiveness and dissemination 2005 http://www.preventionresearch.org 10.1007/s11121-005-5553-y 16365954

[B29] Raaijmakers M Koffijberg H Posthumus J van Hout B van Engeland H Matthys W Assessing performance of a randomized versus a non-randomized study design Contemp Clin Trials 2008 29 293 303 10.1016/j.cct.2007.07.006 17707138

[B30] McCarney R Fisher P van Haselen R Accruing large numbers of patients in primary care trials by retrospective recruitment methods Complement Ther Med 2002 10 63 68 10.1054/ctim.2002.0516 12481953

