# Supplementary material for: Behavioral counseling to prevent childhood obesity – study protocol of a pragmatic trial in maternity and child health care
Source: BMC Pediatr. 2012 Jul 3;12:93. doi: 10.1186/1471-2431-12-93 (PMC3499399; doi:10.1186/1471-2431-12-93)
Supplement: Additional file 1 — Information on “the Smart Family”-exercise and nutrition guidance method. [file 1471-2431-12-93-S1.pdf]

# The Smart Family

## exercise and nutrition guidance method

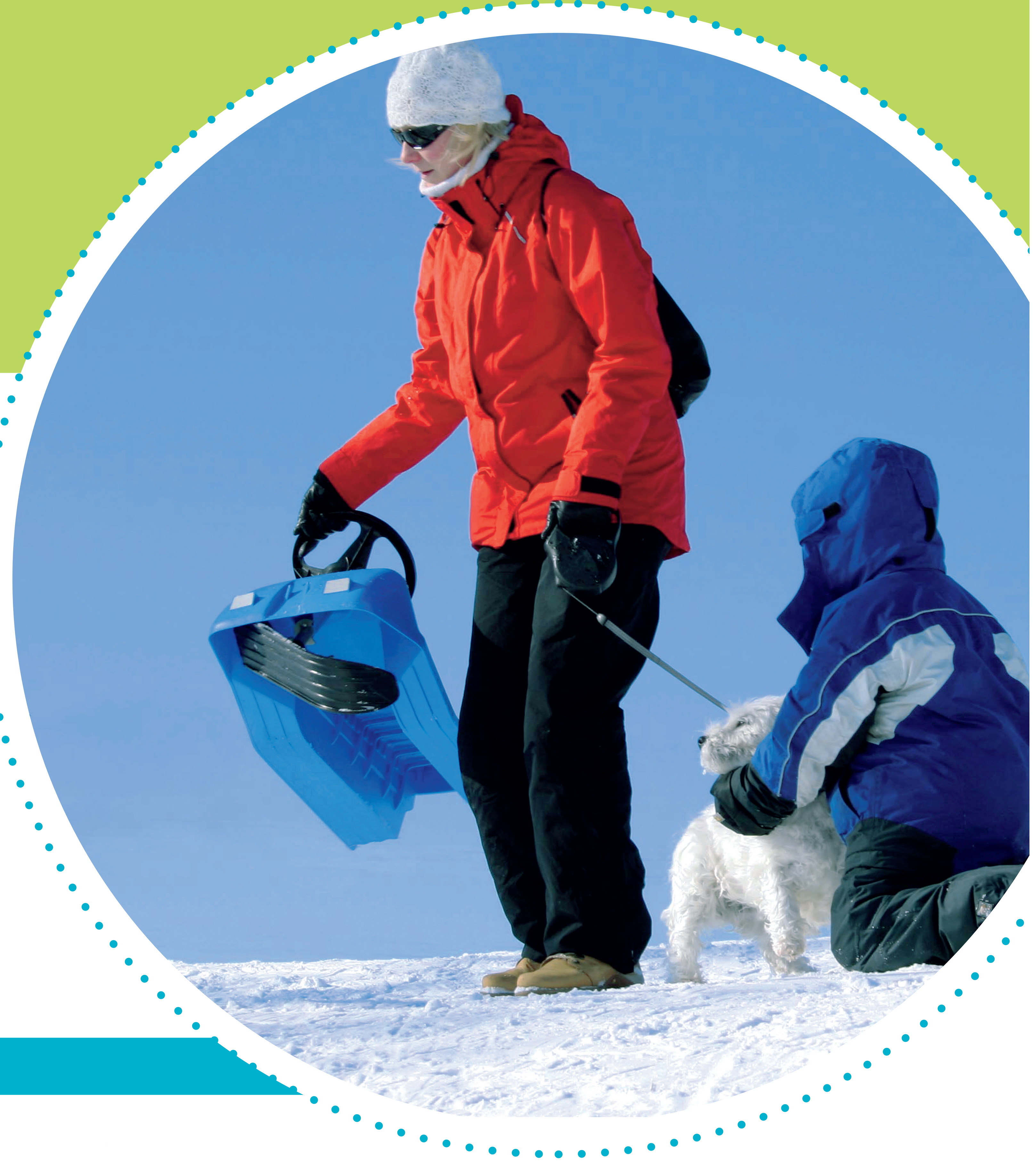

## Transforming recommendations and research results into practical activities

- The Smart Family programme of the Finnish Heart Association aims to promote good exercise and nutritional habits among children and families and to prevent obesity.
- The programme is based on Ministry of Social Affairs and Health recommendations on the activities of child welfare clinics and on an intervention study on the lifestyles of families with children in Turku (STRIP).
- The first phase of the Smart Family programme developed a family-centred exercise and nutrition guidance method to be used as part of the normal activities of maternity and child welfare clinics.
- Child welfare clinics are the easiest way to approach families with children under school age.
- During the second phase of the Smart Family programme, the method will be applied to school health care.

## Family-centred guidance gives good results – working tools for public health nurses

- The tools help public health nurses to respect in their activities the individual needs of the families and to support their resources.
- The method supports and clarifies the role of the public health nurse in providing guidance and makes it easier to bring exercise- and nutrition-related lifestyle matters to the fore.

### The family exercise and nutrition habits card

- The family's card forms the basis of the guidance method.
- The family have the same card throughout their period at the child welfare clinic.
- It includes questions on the mother's, father's and children's exercise and nutrition habits and allows the evaluation of one's own practices, the setting of goals and the monitoring of goal achievement.
- A simple traffic light system makes the answers easy to grasp.

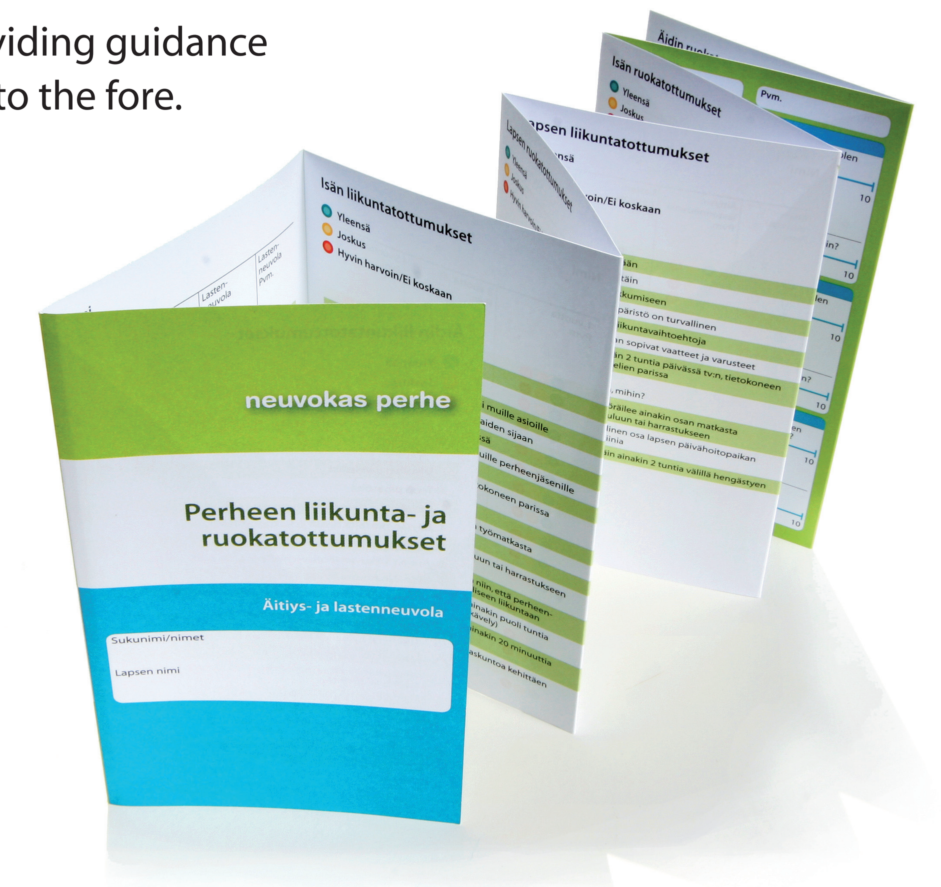


| Example questions relating to the parents' exercise and nutrition habits | Mother | Father |
|---|---|---|
| Exercise makes me feel good | ● ● ● | ● ● ● |
| I exercise at least 5 days a week for at least half an hour, so I get slightly out of breath (for example by brisk walking) | ● ● ● | ● ● ● |
| I have breakfast and at least one main meal every day | ● ● ● | ● ● ● |
| **Example questions relating to children's exercise and nutrition habits** | | |
| The child likes exercise | ● ● ● | |
| The child exercises every day for at least 2 hours and sometimes gets out of breath | ● ● ● | |
| The child has breakfast every day and another 4 to 5 meals or snacks | ● ● ● | |

● (green) = usually
● (yellow) = sometimes
● (red) = very rarely/never

### Information pack

- An information pack for public health nurses, containing recommendations for matters relating to exercise and nutritional habits and some background information.
- Tips for the practical implementation of family-centred guidance methods in exercise and nutrition guidance.

## From a programme to a national guidance method

- The Smart Family guidance method was piloted for six months in ten municipalities.
- The public health nurses who took part in the pilot study thought the guidance method was much needed, useful and a good tool to support their activities.
- The guidance method provided new tools for exercise and nutrition guidance and for the prevention of obesity.
- The aim is to introduce the use of the guidance method at all maternity and child welfare clinics in Finland.

### Year 2011

- The Smart Family -method will expand to primary school health care and smoking will be included as a theme.

### Image pack

- Easy-to-grasp material to support individualised exercise and nutrition guidance.
- The subject matters of the image pack support the questions on the exercise and nutrition habits card.

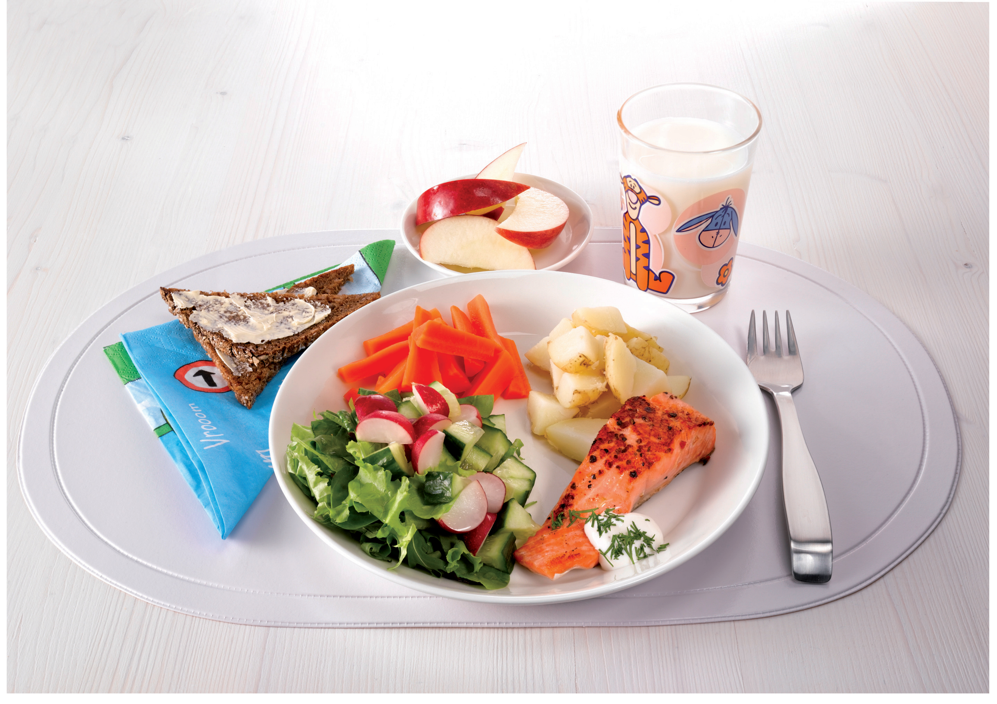

### Continuing education

- For public health nurses; to support and facilitate the adoption of the working tools and the guidance methods.
- Full-day events for continuing education provide an opportunity to practise using Smart Family tools as a part of your normal guidance activities.

**The following organisations took part in planning the Smart Family programme:**

- The Turku University STRIP project
- Ministry of Social Affairs and Health
- Finnish National Public Health Institute
- Development and Research Centre for Maternity and Child Health Care at the National Research and Development Centre for Welfare and Health (STAKES)
- Finnish Union of Public Health Nurses
- Finnish Osteoporosis Association.

The Smart Family programme is part of the Finnish Heart Plan (2006-2011).
The programme was produced with support from Finland's Slot Machine Association and the Ministry of Social Affairs and Health.

**For more information:**
Sydänliitto / Neuvokas perhe t. +358 9 752 752 1
Finnish Heart Association
www.sydanliitto.fi/neuvokasperhe

Suomen Sydänliitto ry
Finnish Heart Association

SUPPORTED BY ray
